# Accounting for Negative Automaintenance in Pigeons: A Dual Learning Systems Approach and Factored Representations

**DOI:** 10.1371/journal.pone.0111050

**Published:** 2014-10-27

**Authors:** Florian Lesaint, Olivier Sigaud, Mehdi Khamassi

**Affiliations:** 1 Sorbonne Universits, UPMC Univ Paris 06, UMR 7222, Institut des Systèmes Intelligents et de Robotique, Paris, France; 2 CNRS, UMR 7222, Institut des Systèmes Intelligents et de Robotique, Paris, France; Georgia State University, United States of America

## Abstract

Animals, including Humans, are prone to develop persistent maladaptive and suboptimal behaviours. Some of these behaviours have been suggested to arise from interactions between brain systems of Pavlovian conditioning, the acquisition of responses to initially neutral stimuli previously paired with rewards, and instrumental conditioning, the acquisition of active behaviours leading to rewards. However the mechanics of these systems and their interactions are still unclear. While extensively studied independently, few models have been developed to account for these interactions. On some experiment, pigeons have been observed to display a maladaptive behaviour that some suggest to involve conflicts between Pavlovian and instrumental conditioning. In a procedure referred as negative automaintenance, a key light is paired with the subsequent delivery of food, however any peck towards the key light results in the omission of the reward. Studies showed that in such procedure some pigeons persisted in pecking to a substantial level despite its negative consequence, while others learned to refrain from pecking and maximized their cumulative rewards. Furthermore, the pigeons that were unable to refrain from pecking could nevertheless shift their pecks towards a harmless alternative key light. We confronted a computational model that combines dual-learning systems and factored representations, recently developed to account for sign-tracking and goal-tracking behaviours in rats, to these negative automaintenance experimental data. We show that it can explain the variability of the observed behaviours and the capacity of alternative key lights to distract pigeons from their detrimental behaviours. These results confirm the proposed model as an interesting tool to reproduce experiments that could involve interactions between Pavlovian and instrumental conditioning. The model allows us to draw predictions that may be experimentally verified, which could help further investigate the neural mechanisms underlying theses interactions.

## Introduction

Persistent maladaptive and suboptimal behaviours are commonly observed in animals, including Humans, and supposed to result from possible constraints (e.g. energy versus efficiency trade-off) solved by the interaction of neural mechanisms not clearly identified yet. Breland and Breland [1] studied animals that learned to retrieve rewards given some action (e.g. drop an object to get food). They observed that, while successful at first, these animals developed strange behaviours which blocked them in achieving the rewarding action (e.g. paws kept clenched on the food-predicting object). Hershberger [2] studied how chicks failed to learn to run away from visible food to eventually get access to it. Guitart-Masip et al. [3] showed that many humans have difficulties to learn to withhold from acting to get rewarded in a go/no-go task. These maladaptive behaviours have been suggested to arise from the interactions between multiple decision systems in the brain [4]–[7], namely Pavlovian and instrumental systems. Pavlovian conditioning is the acquisition of responses associated to initially neutral stimuli that have been paired with rewards while instrumental conditioning is the acquisition of an active behaviour in order to retrieve rewards or avoid punishments. However, the respective mechanisms of these two types of conditioning and how they interact are still unclear.

An example of such maladaptive behaviour was experimentally investigated by Williams and Williams [8], whose initial goal was to explore the properties of the pecks developed by pigeons in procedures subsequently referred as *autoshaping* [9]. A classical autoshaping procedure elicits a standard Pavlovian phenomenon. It consists in pairing a conditioned cue (e.g. a light) with the subsequent delivery of food and results in animals developing robust conditioned responses (e.g. pecks) towards the conditioned cue, even if these responses were unnecessary to be rewarded. Actually, Brown and Jenkins [10] found autoshaping to be a more effective way of getting animals to engage with objects for subsequent instrumental experiments, such as pulling a chain or pressing a lever, than other training protocols. Williams and Williams [8] developed another protocol, that was afterwards referred as a *negative automaintenance procedure*, which consisted in a setup identical to an *autoshaping* procedure, with the exception that pecking the light turned it off and reward was subsequently omitted. Unexpectedly, they observed that most of their pigeons persisted, although to a lower extent, to peck the light despite its negative consequence, losing during the process a significant amount of reward. The phenomenon was further investigated in both pigeons [11]–[14], and other species such as rats [15]–[17] and rabbits [18] with similar results. However, in a more recent study on pigeons with a slightly different negative automaintenance procedure, Sanabria et al. [19] did not observe as much sustained detrimental pecks as observed by Williams and Williams [8], casting a shadow over the original results. While the differences in the procedures might be one reason of such conflicting results, the present paper develops an additional possible reason.

According to multiple studies [4], ]16],[19], negative automaintenance investigates the confrontation between Pavlovian processes and instrumental ones. It is our interpretation that conditioned responses develop because of the contingency between the conditioned stimulus and the reward (Pavlovian conditioning) and one would expect pigeons not to peck as it prevents them from being rewarded (instrumental conditioning). Understanding the underlying neural mechanisms that result in such behaviours is also important to clarify the constraints and strategies developed by years of evolutions for animals to survive in nature.

Killeen [13] and Sanabria et al. [19] have proposed computational models to account for the pecking behaviour described above. However their models are very specific to the task and not easily generalizable to the study of other phenomena. Dayan et al. [4] proposed a more general computational model of interactions between Pavlovian and instrumental conditioning and took negative automaintenance as an illustration, focusing on the first experiment of Williams and Williams [8] that introduces the general phenomenon, but without investigating its subtleties resulting from more specific subsequent experiments.

Initially inspired by this latter model, Lesaint et al. [20] developed a computational model that accounts for a variety of experimental results in rats undergoing an autoshaping procedure [21], especially observed inter-individual variabilities of behaviours within the population. In this study, some rats (sign-trackers) came to approach and engage the conditioned stimulus (CS) itself more and more avidly, whereas other rats (goal-trackers) learned to approach and engage the location of food delivery upon CS presentation, a variability also visible at the physiological and pharmacological level.

In the present study, we show that the model of Lesaint et al. [20], initially developed to account for autoshaping in rats, can reproduce with barely no modifications the experimental data on autoshaping and negative automaintenance in pigeons. Especially, the model suggests as one of the plausible reasons regarding the conflicting data of Williams and Williams [8] and Sanabria et al. [19], that the variability of observed behaviours partially results from the presence of sign-trackers and goal-trackers within pigeons. It is also able to account for other experimental data about the necessary properties of the cues to express negative automaintenance [8]. Moreover, the model generates predictions that may be tested with additional experiments. We further discuss the interest of the combination of concepts on which the model relies for the reproduction of experimental data on Palovian and instrumental conditioning.

## Methods

### Model

**Figure 1:**
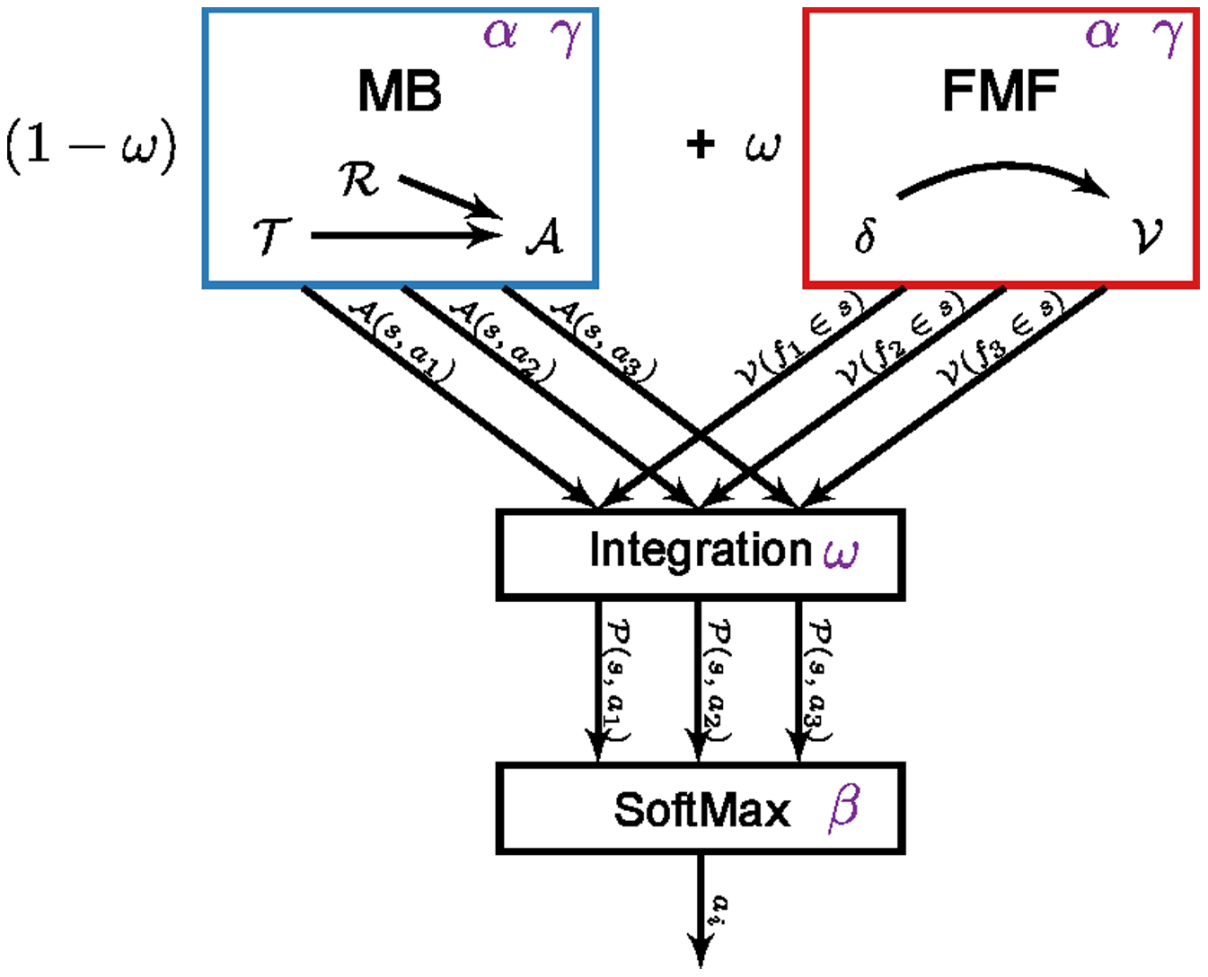
Model used for simulations. The model is composed of a model-based system (MB, in blue) and a Feature-Model-Free system (FMF, in red) which provide respectively an advantage function 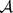 for actions 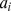 given a state 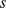 and a value function 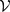 for each feature 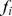 that compose the given state. These values are integrated in 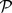, prior to be used into an action selection mechanism. The various elements may rely on some parameters (in purple).

The model from which the present results are generated is described in depth in [20]. Here we describe the computational mechanisms of the model that capture the experimental data in pigeons. The model is based on a reinforcement learning (RL) method, which describes how an agent should adapt its behaviour to rewarding events. Reinforcement learning relies on Markov Decision Processes (MDP) where the environment is described as a set of states between which the agent can move by acting (see next section). The model is composed of two distinct reinforcement learning systems that collaborate, through a weighted sum integration of values respectively computed by each system, to select an action at each step of the experiment (Figure 1) [7]. One system favours rational and optimal plans of actions while the other leads to more impulsive choices.

The first system is a model-based (MB) system that learns the long term consequences of actions by estimating an approximate model of the world (a transition function 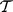 and a reward function 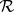) on which to build action plans. The model is sufficient to anticipate the delivery of food subsequently to key lights appearance and therefore the interest of being close to the magazine even before its delivery. It is also sufficient to learn that pecking leads to reward omission and should be avoided. This system produces a goal-directed behaviour [22], [23]. In our implementation of this Model-Based process, the system infers the advantage (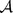) of taking each action in each situation from its model, given the classical following formulae:
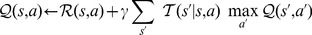
(1)




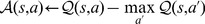
(2)where the discount rate 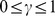 classically represents the preference for immediate versus distant rewards and 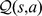 is the expected value of doing action 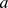 in state 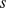 (it corresponds to the discounted accumulation of rewards expected from that moment if subsequently following the assumed best plan of actions). At each step, the most valued action is the most rewarding in the long run (e.g. approaching the magazine to be ready to consume the food as soon as it appears). Equation 1 reflects the prospective process by which the simulated agent estimates the future consequences of performing action 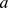 in state 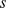. If action 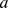 is assumed to lead to a reward 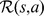 or with a good probability 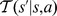 to another state 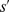 with a high quality action 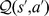 then the agent will associate a high 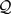-value to the state-action pair 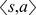. Equation 2 deduces the advantage of performing action 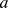 in state 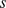 by comparing its 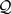-value with the maximal possible 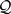-value of all available actions in the same state. Note that other implementations could be possible.

The second system is model-free (MF). It does not learn an internal model of the world but incrementally learns to associate values to features of the environment, favouring actions towards valued ones. As a result, this system produces a reactive behaviour in a way similar to habits [24], [25]. Without an internal model, it cannot consider the consequences of an action and hence solely bases its decision on the a priori expectation values it learns.

In traditional RL (e.g. the MB system), values are learned over abstract states (e.g. arbitrarily defined as *s*_1_, *s*_2_… *s_x_*), such that similarities between situations (e.g. presence of a magazine) are ignored. The present system learns values (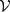) over features (e.g. food, lever or magazine) and is further defined as the feature model-free system (FMF). Using features reintroduces the capacity to use and benefit from similarities between states. The incremental learning of values relies on a reward prediction error (RPE) signal 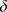, and works as follows:
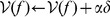
(3)

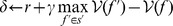
where 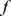 is the feature that has been focused on by the action 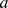 in state 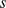. The max suggests that all the features 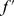 of the new state 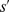 are considered and the most valued one is used to compute the RPE, even if it might not be the feature focused by the next chosen action. This update rule (Equation 3) may be paralleled with the one of the classical Model-Free 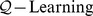 algorithm [26] where 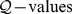 are used in place of 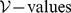. While very similar, such rules can actually produce very different results and patterns depending on the involved situations. The model embeds a feature-function 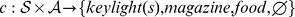 that returns the feature the action 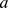 was focusing on in state 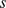 (e.g. it returns the key light when the action was to engage with the key light). In [20] we hypothesized that, similarly to classical model-free systems, 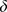 parallels the phasic dopaminergic activity (DA) [27]. This signal enables to revise and attribute values, seen as motivational or incentive, to features without the need of the internal model of the world used by the MB system. When an event is fully expected, there should be no RPE as its value is fully anticipated; when an event is positively surprising, there should be a positive RPE [28]. The values learned bias the behaviour towards actions that are directed towards the most motivational features (e.g. engaging with the key light would be biased by the general motivational value of the key light). This might lead to favour suboptimal actions with regard to maximizing rewards (e.g. engaging with the negative key light prevents pigeons from being rewarded). The FMF system models the attraction developed by reward-predicting stimuli in such experiments, i.e. incentive salience [29]–[31].

The model does not base its decision on a single system at a time. Rather, the values of the MB system (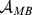) and the FMF system (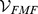) are integrated such that a single decision is made at each time step. The values computed by these two systems are combined through a weighted sum and transmitted to a softmax action selection mechanism that converts them into probabilities of selecting actions given a situation (Figure 1). The integration is done as follows:
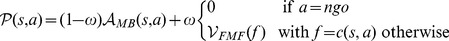
(4)where 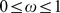 is a combination parameter which defines the importance of each system in the overall model. Pigeons may be modelled with a particular 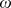 value, different 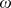 values producing different characteristics of behaviour. The integration (Equation 4) differs from the one suggested by Lesaint et al. [20] as the tasks presented here introduce the new notion of *refraining from engaging*. We hypothesize that refraining from engaging with a stimulus does not benefit from the FMF bonus associated with such stimulus, hence the *a* =  *ngo* condition in the second part of the equation. This hypothesis is based on studies of go and no-go learning [3], [32] that suggest the presence of a bias for engaging against withholding. Note that this modification could be propagated to the previous studies [20], [33] without any impact. Indeed, the experiments already accounted for by the model do not require to refrain from acting.

The model incrementally learns from experience at each step. FMF and MB systems are updated according to the action 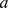 taken by the full model in state 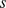 and the resulting new state 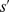 and retrieved reward *r*.

### Task modelling

**Figure 2:**
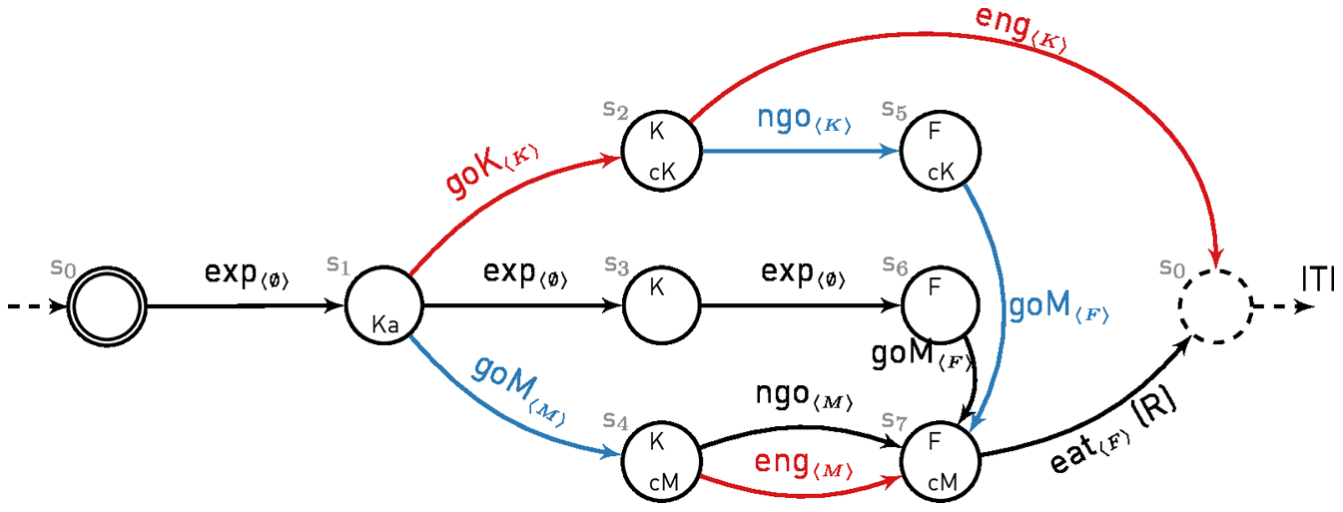
Computational representation of the negative automaintenance procedure. MDP accounting for Experiment 1 in Williams and Williams [8] and for the Brief PA protocol of Sanabria et al. [19]. States are described by a set of variables: *K*/*F* - negative Key light/Food is available (Magazine is always available, hence it is not shown), *cM*/*cK* - close to the Magazine/negative Key light, *Ka* - Key light appearance. The initial state is double circled, the dashed state is terminal and terminates the current episode. Actions are engage (eng) or refrain from engaging (*ngo*) with the proximal stimuli, explore (exp), or *go* to the *M*agazine/*K*ey light and *eat*. Only the *eat* action is rewarded (R), such that in this experiment, pigeons that engage with the key light receive nothing during the trial. For each action, the feature being focused on is displayed within brackets.

**Figure 3:**
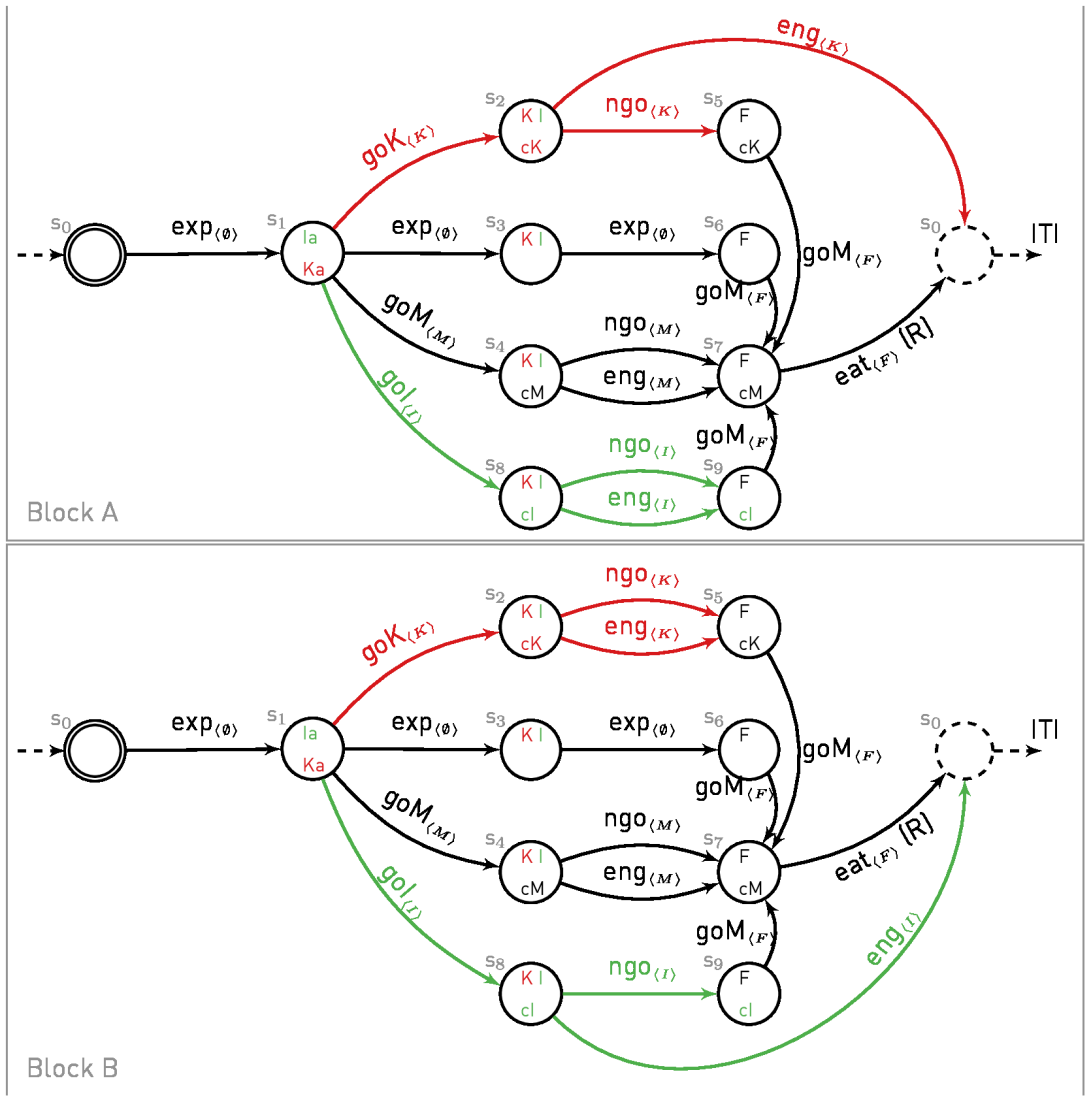
MDP for simulation of Experiment 3 in Williams and Williams. Legend is as in Figure 2. The path involving an engagement with the negative key light is highlighted in red. A new irrelevant key light (green), the associated paths and actions are added to the MDP of Figure 3. The animal starts in block A. During the experiment, blocks can be switched without informing the animal, such that the contingencies are reversed between keys.

**Figure 4:**
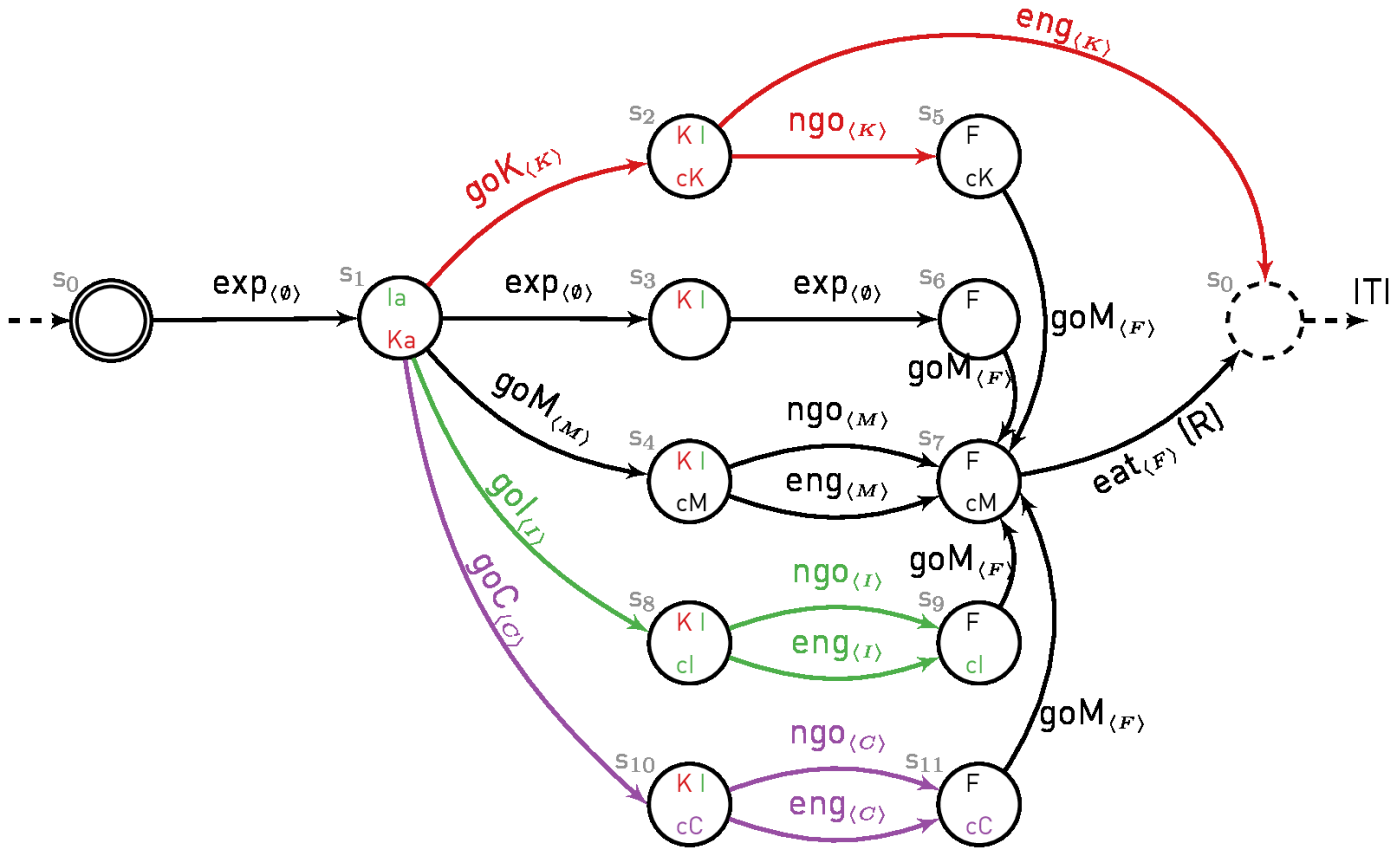
MDP for simulation of Experiment 4 of Williams and Williams. Legend is as in Figure 3. A new continuous irrelevant key light (purple), the associated paths and actions are added to MDP of Figure 3 (Block A). Note that while not shown, as for the Magazine, the Continuous key light is present in all states. Paths are activated/deactivated depending on the current phase of the current protocol (Table 1).

Figures 2, 3 and 4 show the MDPs used to simulate the different experiments of Williams and Williams [8] and Sanabria et al. [19]. We assume that each experimental trial can be simulated with a finite horizon episode, that is by a single run in an MDP with an initial and a terminal state. Furthermore, to comply with the MDP framework, we assume that engagement is necessarily exclusive to one or no stimulus and we do not model time, which is sufficient to replicate the experimental data.

In Experiment 1 (Figure 2), the agent starts from an empty state (*s*_0_) where there is nothing to do but explore. At some point the key light is turned on (*s*_1_). The agent can either approach the key light (*s*_2_), approach the magazine (*s*_4_) or keep exploring (*s*_3_,*s*_6_). If close to the key light (*s*_2_), it can either engage with it which ends the trial without reward (*s*_0_), or refrain from engaging until food is eventually delivered (*s*_5_). If close to the magazine (*s*_4_), engaging or not has no impact and leads to food delivery (*s*_7_). Finally, if the agent is far from the magazine (*s*_5_,*s*_6_), it first needs to get closer (*s*_7_) before consuming the food, hence retrieving the only available reward in this trial (R). It ends in an empty state (*s*_0_) which symbolizes the start of the inter-trial interval (ITI): no food, no lever and *an empty but still present magazine*. Paths in red are those that should be favoured by the FMF system, leading to the potentially detrimental action of engaging with the key light. Paths in blue are those that should be favoured by the MB system, successfully leading to reward delivery.

Experiments 3 and 4 use additional key lights (irrelevant and continuous). Each light extends the previous MDP with an additional path as described in Figures 3 and 4. The main idea is that animals can orient towards any key light (or magazine) and subsequently engage with it. Based on the simulated protocols, paths can be activated/deactivated during experiments, such that only available actions are considered by the model in its decision. In Experiment 3, the role of the keys (K and I) are reversed multiple times during the experiment (Blocks A and B in Figure 3).

In Williams and Williams [8], the key light is immediately turned off following a peck. In Sanabria et al. [19] protocol, the key light is maintained for a fixed period, whatever the behaviour of the pigeon. Food is then only delivered if no contacts with the key light are made during that period. Pigeons could therefore produce multiple pecks during a trial, hence the difference in scales between both studies that is not replicated in our results. Despite such difference in protocols, the MDP of Figure 2 is also used to simulate the results by Sanabria et al. [19]. Consequently, we mainly explain the difference of behaviours between the two studies by an inter-individual variability in pigeons, simulated by different parameter values, rather than by the difference in protocols.

#### Inter-trial interval (ITI)

While the MDP does not model the ITI, we assume that the presence of a stimulus (key light or magazine) during ITI degrades its values in the model. This current hypothesis is simulated by revising the values of the magazine and the continuous key light (if available) with the following formulae:
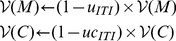
(5)where the parameters 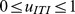 and 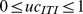 reflect the impact of the presence of the magazine and the continuous key light during ITI on their acquired value in the FMF system. A low value symbolizes a low impact and therefore a low revision of the value associated to the stimulus.

Note that extending the MDP with a set of states to represent this interval would have increased the complexity of the MDP, introduced non-Markov aspects to the task and increased the time required for simulations. Furthermore, while it might have led to the same results, the interpretation would have been different from our hypothesis, as downgrading the values would have required engagement and not only the presence of stimuli.

#### Pre-training

No MDP was used to simulate the possible autoshaping pre-training that underwent some of the pigeons in the experiments, nor the necessary familiarization with the Skinner box and the magazine mechanism. Rather, we initialize the model with values (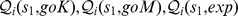) that simulate the action-values acquired during such pre-training phases.

These values have no impact in the long run behaviours as they are revised by incremental learning during the simulation. They mainly help in reproducing the initial tendencies of pigeons to interact with the experimental environment.

### Model parameters and simulations

The model relies on a set of 8 parameters (a shared learning rate, a shared discount rate, a selection temperature, an integration parameter and 3 initial conditions) that need to be tuned for simulations to reproduce experimental data. The parameter values used were obtained by hand tuning. More automatic tuning methods (e.g. fitting optimisation algorithms [20]) were not possible without more precise numerical experimental data. Hence we only tried to qualitatively replicate the experimental results of Williams and Williams [8] and Sanabria et al. [19].

Nevertheless, simulation results were generated with a single set of parameter values for all experiments of Williams and Williams [8] and Sanabria et al. [19], with the exception of 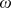 and 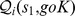 (see Table 2). Following the terminology used in Lesaint et al. [20] to categorize rats, we can say that we simulated *sign-trackers* (high 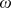) and *goal-trackers* (low 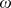) pigeons.

**Table 2:** Parameters values used for simulations.

Pigeons	Grp	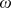	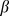	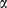	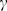	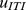	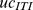	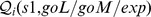
Williams and Williams *	STs	0.9	0.15	0.2	0.9	0.3	0.2	0.0/0.2/0.2
Sanabria et al.	GTs	0.2	0.15	0.2	0.9	0.3	0.2	0.8/0.2/0.2

Parameter values used to replicate studies from Williams and Williams [8] and Sanabria et al. [19], with their interpretation: goal-trackers (GTs) or sign-trackers (STs). * Note that one pigeon of Williams and Williams (P19) behaved as those of Sanabria et al. (i.e. it would be simulated with GTs parameters).

Varying the 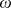 parameter is sufficient here to reproduce the experimental results. This was done here for parsimony, in order to highlight the key important mechanisms to explain experimental data without giving the model too many degrees of freedom. It is however almost certain that pigeons would not share the exact same parameter values in reality. Especially, breeding procedures, housing procedures and training procedures might have some impact on the averaged neural mechanisms properties modelled with these values.

Sanabria et al. [19] pigeons were divided into multiple groups that underwent different protocols, with multiple mixed phases of positive and negative training. Except for 3 pigeons, Williams and Williams [8] did not train their pigeons on the key lights before the main experiments. For a better comparison between these studies, we only focus on the pigeons of Sanabria et al. [19] that were briefly exposed to autoshaping before being confronted to negative automaintenance (*Brief PA* protocol) and pigeons with no pre-training in Williams and Williams [8], hence the difference of value for the 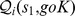 parameter.

## Results

We applied the present model to the various MDPs to replicate the results of Experiments 1, 3 and 4 of Williams and Williams [8] and also to some results of Sanabria et al. [19] (*Brief PA* protocol).

### Classical negative automaintenance

The central phenomenon that we intend to replicate with the present computational model is the greater or lesser persistence in pigeons to peck a key light that, while predictive of reward delivery, leads to its omission in case of contact.

**Figure 5:**
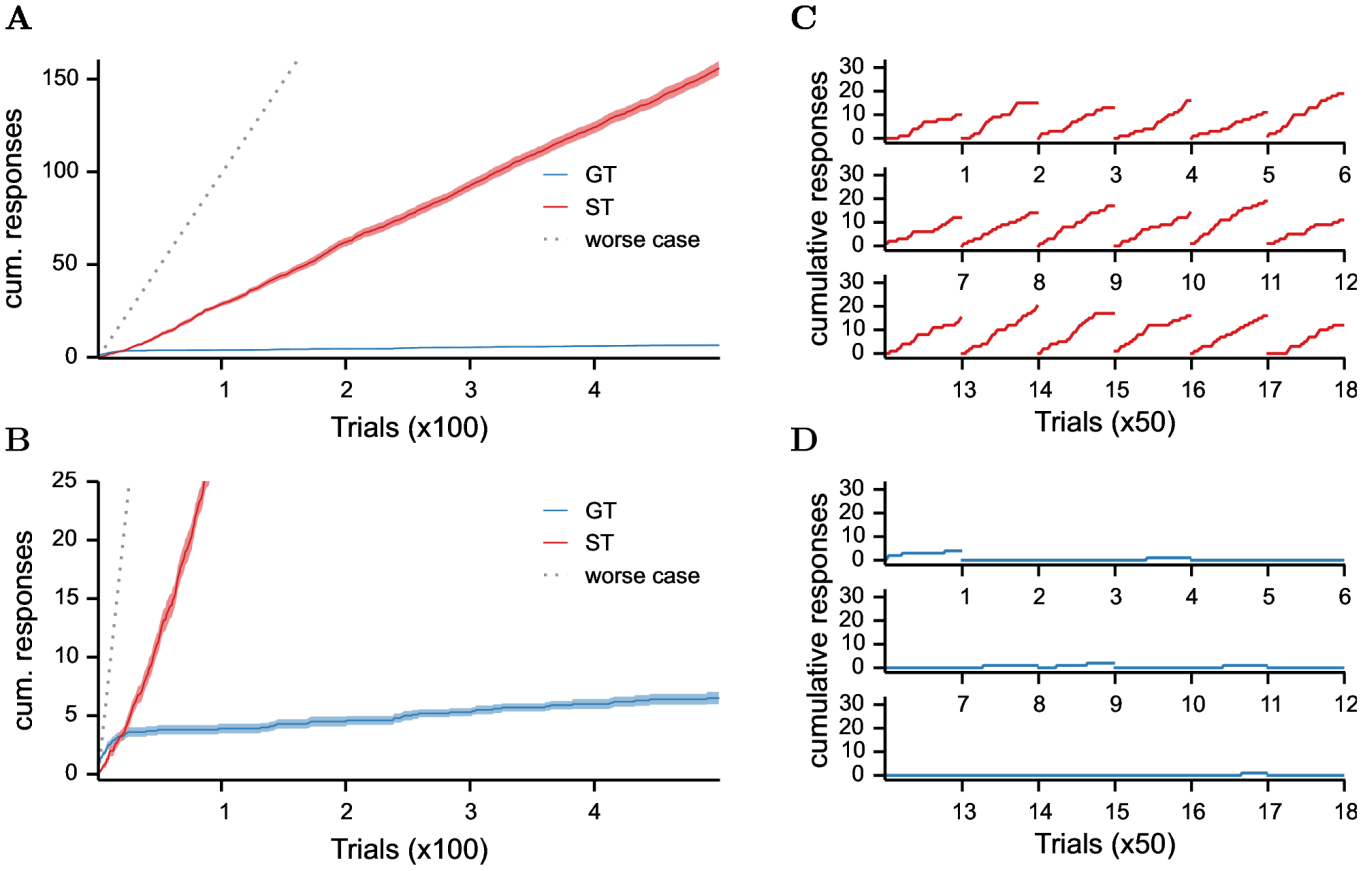
Simulation of Experiment 1 of Williams and Williams [8] and Brief PA protocol of Sanabria et al. [19]. (**A**) Cumulative pecks towards negative key light made by 8 simulated GT pigeons (blue curve) and 8 simulated ST pigeons (red curve). The dotted grey curve simulated the worse case scenario (if pigeons would have pecked at every trials). Data are expressed as mean ± SEM. (**B**) Zoom of (A) for a better reading of the blue curve (GTs). (**C**) Cumulative pecks for one ST pigeon by blocks of 50 trials. To be paralleled with Figure 1 of [8]. (**D**) Cumulative pecks for one GT pigeon by blocks of 50 trials.

In the first experiment of Williams and Williams [8], pigeons undergoing a negative automaintenance procedure failed to completely stop pecking at the key light such that they missed a consequent number of rewards. Only one pigeon (P19) retrieved more than 90% of the available rewards. The model can replicate the general behaviour of all other pigeons with one set of parameter values, and P19 with a different set of values. The red curve in Figure 5 shows pigeons that are unable to refrain from pecking and lose almost half of the 50 possible available rewards per session. This behaviour persists over time.

In a more recent study, Sanabria et al. [19] challenged these results of Williams and Williams [8] as they ran a similar experiment but observed a significant decrease in the detrimental pecks at key light (similar to P19, which was assimilated to a pigeon of Sanabria et al. [19] in simulations). They claimed that remaining pecks did not differ significantly from those that can be observed after a classical extinction procedure. Actually, in an extinction procedure, the conditioned key light is subsequently decorrelated from food delivery, which results in pigeons stopping to emit conditioned responses, except from few exploration pecks. The model is also able to replicate such results using the same MDP despite a slight difference in the experimental protocols. The blue curve in Figure 5 shows pigeons that start to peck (by exploration or familiarization) but quickly learn to refrain from pecking to retrieve rewards. We would consider P19 as part of such pigeons.

Each time a simulated pigeon does not peck the key light, its motivational value is reinforced as the key light is contingent to reward delivery (Figure 2). This naturally increases the tendency, promoted by the FMF system, to peck during subsequent trials. As in Lesaint et al. [20], we assume that the presence of the magazine during ITI makes it lose parts of its acquired motivational values (A low 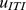), hence the magazine remains less attractive than the key light and the pigeon never really focuses on it while key light is active. The relative attractiveness of the key light is however balanced by pecks, as the omission of rewards produces a decrease in the key light motivational value.

The MB system solves the task by finding the shortest sequence of actions until reward. As a result, it favours approaches to the magazine, as this is the shortest path to reward (Figure 2). Note that other paths would only delay reward delivery by one step and hence are still positively evaluated (especially with a high 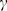). When close to the key light, it strongly favours refraining from pecking, as this would prevent delivery of the subsequent reward.

To summarize, in the MB system, the values of all actions but engaging with the key light increase until a convergence level, which depends on how short is the following optimal path to reward. The values then remain at that level until the end of the experiment. The value of engaging the key light remains to 0 as it leads to no reward. In the FMF system, the lever acquires a value that keeps oscillating around a certain level, decreasing at key pecks and increasing otherwise. The magazine value increases at each trial but is partially reset during ITI, such that its value remains at a low level.

When the model gives a high influence (large 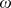) to the FMF system in the decision process, it produces pigeons that persist in pecking. The FMF system introduces a bias towards actions that lead to approach and interact with stimuli that acquired motivational values, in this case the key light. The resulting low influence of the MB system cannot compensate for this bias. This leads to the production of the expected maladaptive behaviour observed in Williams and Williams pigeons, except for pigeon P19 (Figure 5, red curve).

When the model gives a low influence (small 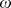) to the FMF system in the decision process, it produces pigeons that quickly learn to stop pecking after a few exploration pecks. Indeed, the MB system favours behaviours that maximize cumulation of rewards, that is behaviours that do not lead to peck the key light. Pecks observed in such simulated pigeons are mainly due to exploration. The FMF system is not able to bias the actions enough to lead to a maladaptive behaviour and pigeons stop pecking as in Sanabria et al. [19] study and for pigeon P19 of Williams and Williams [8] (Figure 5, blue curve).

Given the provided equations, refraining from pecking does not completely compensate for a prior peck and vice versa. Combined with exploration, this mechanism leads to oscillations of the behaviour of pigeons that are not a perfect alternation of pecks and abstentions. Hence, from time to time, pigeons will stop pecking, start accumulating food, and by this process reinstate the attractiveness of the key light and the resulting subsequent detrimental pecks.

Thus, the current model is able to account for these, at first sight, contradictory results. With different parameter values (see Table 2), the model can reproduce pigeons that fit those of Williams and Williams [8] and those of Sanabria et al. [19]. It explains the difference between their findings as a result of a possible interindividual variability in pigeons. Some are more prone to rely on the FMF system to guide their behaviours while others rely on the MB system. We can define the pigeons of Williams and Williams [8] as being mainly sign-trackers and those of Sanabria et al. [19] as being goal-trackers.

It is important to note that the model describes the significantly lesser amount of reward received by sign-trackers relative to goal-trackers as a consequence and not a cause of their behaviour (simulated by a different 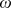 parameter).

### Avoidance strategies

Experiment 2 of Williams and Williams [8], using a different protocol, only controlled that key lights had to be contingent to some rewards to produce key pecks and was not simulated. In their Experiments 3 and 4, Williams and Williams [8] further investigated the properties of the sustained pecks, especially if they could be oriented to alternative keys with different contingencies (avoidance strategies). A model accounting for negative automaintenance should reproduce these properties.

**Figure 6:**
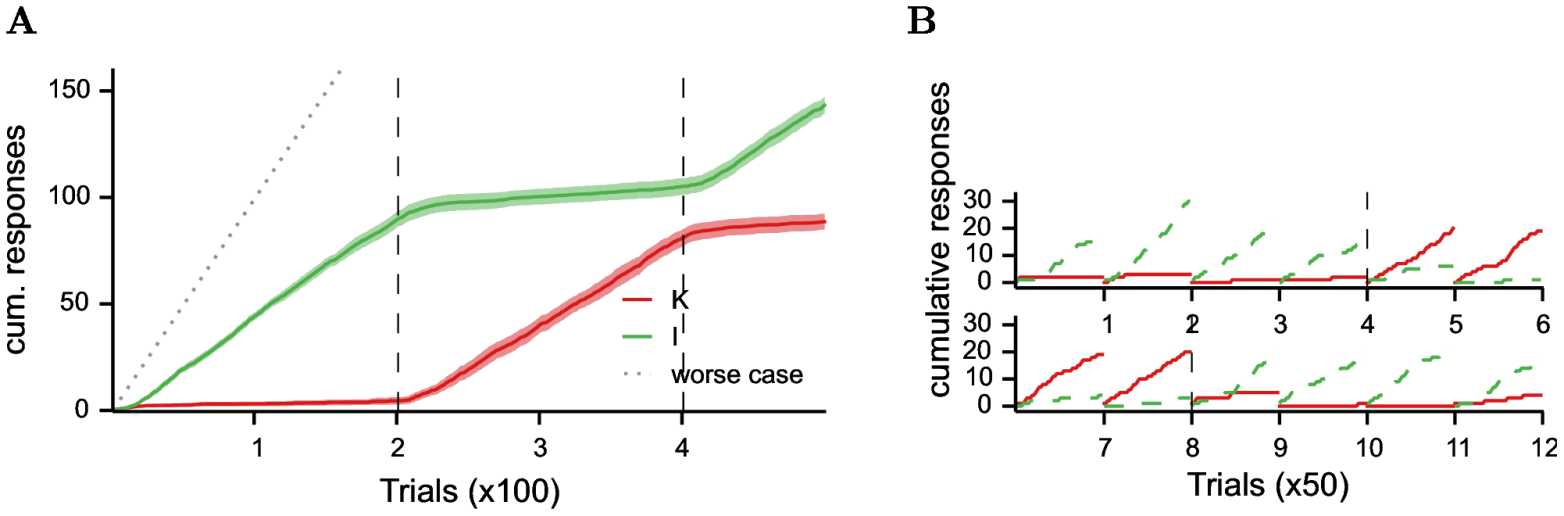
Simulation of Experiment 3 of Williams and Williams [8]. (**A**) Cumulative pecks towards negative key (red curve) and irrelevant key (green curve) over time made by 8 simulated pigeons. Vertical bar indicates reversals of effects between key lights. The dotted grey curve simulated the worse case scenario (if pigeons would have pecked the negative key at every trials). Data are expressed as mean ± SEM. (**B**) Cumulative pecks for one pigeon by blocks of 50 trials. To be paralleled with Figures 5 and 6 of [8].

In Experiment 3, Williams and Williams [8] extended the protocol with an additional key light. The new key light would turn on and off at the same time as the previous one, but pecks would have no effect on it, hence named irrelevant key (I). While it seems that pigeons are unable to refrain from pecking, they are still able to orient their pecks towards the less prejudicial target. They observed that in such procedure, a tendency to peck also developed in pigeons, but favouring the irrelevant key, hence maximizing accumulation of rewards. Furthermore, to study if such tendency could be revised once trained, the effect of keys (K and I) was reversed at some point without informing the pigeon, i.e. pecks at the irrelevant key blocked reward delivery and pecks at the negative one were without effect. They observed that pigeons quickly learned to switch to the new irrelevant key (see Figures 5 and 6 of Williams and Williams [8]).

With the same parameter values used to simulate Experiment 1 of Williams and Williams [8], the model is able to reproduce such properties (Figure 6). Simulated pigeons learn to focus on the irrelevant key (I), learn to avoid the negative key (K), and after an unexpected reversal (I becoming negative and K becoming irrelevant), quickly learn to reverse their behaviour.

The irrelevant key provides pigeons with an alternative path, that is more favoured by the model. The rational MB system favours equally well approaches towards the irrelevant and negative keys as there exists a subsequent path of equal length to reach rewards (classical reinforcement learning theory). Hence, the action selected ultimately depends on the bias introduced by the second system. The FMF system gives a higher value to the irrelevant key relative to the negative one, as the irrelevant key is always contingent to reward whereas the negative key is only contingent to reward when no pecks are performed. As a result, orienting towards the irrelevant key has a higher probability of being chosen.

The effect of reversal is better explained through a concrete example. Assuming that the key light K is negative in the current block *i*, then 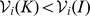 (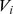 denotes the value during block *i*). When switching to block *i*+1, I becomes irrelevant and 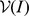 quickly lowers to the level of 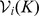 while 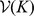 eventually increases to the level of 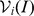, such that after few trials, 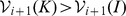. The preferred key alternates between each blocks. Hence, the model nicely explains why pigeons cannot refrain from pecking but are still able to orient pecks to a less detrimental key.

In Experiment 4, Williams and Williams [8] extended the protocol with another additional key light. The new key light would never turn off and pecks would have no effect on it, hence labelled continuous key (C). Note that while always lit on, the position of the key (left/right/middle of the key lights panel) was switched after each trial, such that contrary to the fixed magazine, shifts in its position were predictive of a new possible reward. They studied the relative power of the three keys to attract pecks by combining a subset of them and activating them at different times in different protocols (see Table 1).

**Table 1:** Experimental setups for Experiment 4.

Protocol	Phase 1	Phase 2	Phase 3
A	K	K + C	C
B	K	K + C + I	C
C	K + C	K + C	C
D	K + C + I	K + C + I	C

Lists of keys activated during the different phases of protocols used in Experiment 4 of Williams and Williams [8]. K stands for the negative key, I for the (intermittent) irrelevant key and C for the continuous (irrelevant) key.

**Figure 7:**
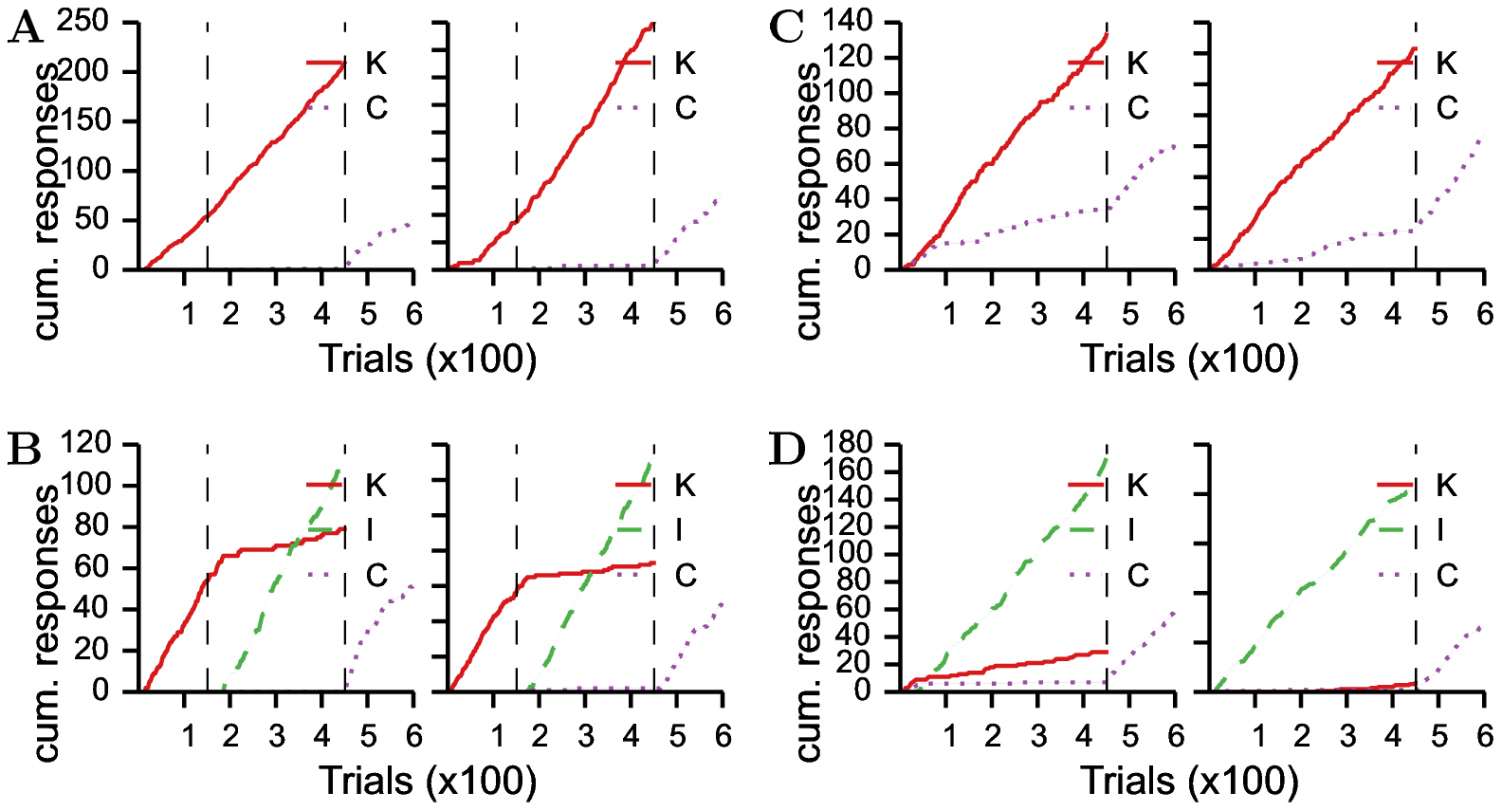
Simulation of Experiment 4 of Williams and Williams [8]. Cumulative pecks towards negative key (solid line), irrelevant key (dashed line) and continuous key (dotted line) over time made by 2 simulated pigeons in different protocols (described in Table 1). Vertical bar indicates phase switches. To be paralleled with Figure 7 of Williams and Williams [8].

They observed that all keys, presented alone produced sustained pecks. The continuous key was ineffective in attracting key pecks when an alternative key, either negative (Figure 7 A and C in Williams and Williams [8]) or irrelevant (Figure 7 B and D in Williams and Williams [8]) was presented. As in Experiment 3, the irrelevant key was effective in attracting away pecks from the negative key (Figure 7 B and D in Williams and Williams [8]).

The model is also able to explain these additional results (Figure 7). The effectiveness of the irrelevant key to attract key pecks has already been explained for Experiment 3. The ineffectiveness of the continuous key results from its presence during ITI. We hypothesize that the presence of a stimulus within the ITI leads to a decrease of its motivational value. Hence, the motivational value of such a stimulus is lower than those of the alternative keys that are time-locked to reward delivery. Note that for the continuous key to be the focus of pecks when presented alone, its motivational value should however remain higher than the value of the magazine. We do not use the same parameter value to decrease the value of the magazine and the value of the continuous key. A variability in the last parameter could explain why in the experimental data, some pigeons did not engage with this continuous key even presented alone.

## Discussion

We applied the model of Lesaint et al. [20] to a new set of experimental data on a negative automaintenance procedure and showed that it is able to qualitatively reproduce different properties of the resulting phenomenon. This model also provides a plausible explanation, although maybe partial, for the conflictual observations between the studies of Williams and Williams [8] and Sanabria et al. [19]. It suggests that negative automaintenance arises from the competition of two reinforcement learning systems, one of which relies on factored representations to use values over features rather than states.

### Pavlovian and instrumental interactions

In [20], the computational model was used to account for a phenomenon described as only Pavlovian, hence one could see both systems as different mechanisms of Pavlovian conditioning [34]. Here, the same model is used to account for a Pavlovian and instrumental interaction phenomenon and systems are rather seen as each accounting for a different type of conditioning [4], [35]. Hence, while using a similar Model-Based system for both studies, it might actually reflect different systems in the brain which would rely on similar principles. It is actually unclear if the whole behaviour of rats undergoing autoshaping, from approach to consumption-like engagement, should be classified as purely Pavlovian [36]–[38]. Further experiments (e.g. outcome devaluation) should be conducted to clarify this point. Extending from studies on how Pavlovian conditioning affects instrumental tasks [35], [39] and studies on how instrumental conditioning can also subsequently affect Pavlovian tasks [40], [41], we suggest that many conditioning tasks might present both Pavlovian and instrumental aspects, with one possibly masking the sparse presence of the other.

In the present case, a parallel can be made between Pavlovian conditioning versus instrumental conditioning and the FMF system versus the MB system. Pecks towards key lights arise because of the values they acquire within the FMF system. These motivational values developed solely by contingencies of key lights with food delivery, independently of actions taken. Hence, the FMF system is at the heart of the Pavlovian aspect in simulated pigeons. It biases their actions towards attractive and predictive stimuli, possibly leading to impulsive, and possibly detrimental engagements. Refraining from pecking, on the other side, is learned by the MB system as the appropriate action to get rewarded. Hence, animals know how to act to optimize their rewards. Therefore, the MB system is at the heart of the instrumental aspect of the behaviour of pigeons. It allows them to learn, to some extent, that they must refrain from acting to retrieve food in specific situations, in this case from pecking. We do not state that instrumental conditioning is Model-Based nor Pavlovian conditioning is Model-Free. It has been shown that both aspects are present in both type of conditioning [34], [39], [42]. In the present work, only the Model-Based aspect of instrumental conditioning and the Model-Free aspect of Pavlovian conditioning are sufficient to replicate the data.

The computational model explains the behaviour of pigeons as a combination of both systems. Each system provides valuation informations regarding the current situation, which are further integrated to eventually determine the action to be taken. Moreover, information is not weighted equally but through a pigeon specific weight (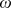) such that one system can have to assess a situation as very detrimental to compensate for the weak positive valuation of this situation attributed by the other system, and avoid a maladaptive behaviour. This is exactly what happens in the negative automaintenance procedure, as the Pavlovian system records the key light as strongly motivational, whereas the instrumental system records any engagement as detrimental. Furthermore, the procedure is such that applying the strategy favoured by one system subsequently reinforce the strategy favoured by the other one. As a result, no system can forever be dominant.

While we currently modelled our integration of MB and FMF systems with a fixed 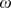 parameter, it might be possible, as suggested in the work of Dayan et al. [4] that such weighting parameter would fluctuate over time based on some yet unknown and still debated criterion [43]–[45]. However, we would still expect that subgroups of individuals would show different parameter values and/or that such values would fluctuate differently. The currently investigated data on pigeons cannot rule out an alternative interpretation that, based on a dynamically computed score (e.g. the difference of estimated uncertainty of each system [43]), only one system might be active and guide the behaviour at a time. However, based on the data about rats undergoing autoshaping experiments simulated with the same model [20], the full spectrum of observed behaviours ranging from STs to GTs [46] and the consumption-like engagement of both STs and GTs, explained by the permanently active FMF system, argues against it.

Interestingly, the current model does not necessarily imply that the two systems would favour conflicting policies. For example, in the case of autoshaping [20] no rewards are lost while the policies favoured are different. Furthermore, the system could even lead to a fruitful collaboration if both systems would favour the same actions, possibly increasing the rate at which the animal would engage with some object and be rewarded accordingly (e.g. in general Pavlovian-to-Instrumental Transfer procedures [3], [37], [47]). We assume that these systems developed for collaboration rather than competition, as negative automaintenance is not really common in a natural environment. One system provides a rational plan of actions while the other offers the opportunity to accelerate it (e.g. reacting at the shadow of a prey rather than waiting for the prey to be entirely visible). Further investigations will be required to determine whether the collaboration between these systems better explains a variety of animal conditioning behaviours than competition.

### Factored representations

Taking advantage of features that compose the environment is not new in the study of Pavlovian conditioning [48]–[53]. It is indeed central to account for phenomena when conflicts arise from the presence of multiple stimuli (e.g. blocking [54] or overexpectation [55]). However, the computational models accounting for Pavlovian conditioning phenomena are usually not relying on the classical RL framework (e.g. MDPs or temporal discounting). Furthermore, they mainly tend to describe the varying intensity of a unique conditioned response rather than the variations of observed responses and they do not explain how an agent can learn sequences of actions.

In traditional studies of instrumental tasks, working at the state level is sufficient to reproduce and explain behavioural data [4], [43], [44], [56]. Tasks are defined as standard MDPs, and classical algorithms cannot use the underlying structure to generalize updates to states that share similarities. These models are mainly used to study learning phases and adaptive capabilities in a changing environment, when animals behave near optimally. Classical algorithms are proven to converge to the optimal solution [26]. In the current task, without relying on very distinct sets of possibly unusual parameter values, two classical algorithms combined in a model would eventually reach the same optimal policy and hence would fail to explain the variability of observed maladaptive behaviours [20].

Here factored representations used in one of the two simulated systems but not the other enable these systems to propose different complementary decisions and thus to explain the variety of behaviours observed in the data. Such factored representations are already present in the RL literature and mainly used to overcome the curse of dimensionality [57], i.e. standard algorithms do not scale well to high dimensional spaces and require too much physical space or computation time. Value function approximations [56], [58], [59] or factored reinforcement learning [60]–[62] help to build a compact value-function or infer the value of states from values of features. These algorithms are only meant to optimize computations but should not produce outputs that diverge from traditional flat RL algorithms. Here, we use factored representation in a different way and make values over features compete in the choice for the next action. The FMF algorithm generates an output different from traditional RL systems.

The capacity of the model to replicate the maladaptive behaviour of pigeons under negative automaintenance results from the difference between the policies developed by the MB system and the FMF system. Such difference is due to the way factored representations are used by the latter system. While the MB system associates value to general situations (states) and favours an optimal policy, the FMF system associates value to salient stimuli (features) biasing actions towards them and favours a different sub-optimal policy (w.r.t. the MDP). The FMF system develops an impetus towards triggering low-level ingrained Pavlovian behaviours towards these salient stimuli as soon as they are presented within a context associated with reward value [4]. In other words, the FMF system and the MB system use different heuristics (paying attention to the situation versus paying attention to salient elements) to guide behaviour. Once combined, these systems conflict in the current experimental setup leading to the observed maladaptive behaviour.

It might be possible to use a factored implementation of the MB system. In such case, we would assume that this system would still assess situations rather than stimuli individually. Hence, it would use factored representations in a traditional way, for computational optimization purposes that should not change the resulting output of the system.

The capacity to attribute values to features also provides a straightforward explanation for why the irrelevant key light attracts most of the pecks in the presence of the negative key light and/or the continuous key light, and why the negative key light attracts most of the pecks in the presence of the continuous key light. Having values over key lights allows for a direct comparison, the development of a preference towards the most valued one, and after its removal, a quick shift towards the second most valued one. By using factored representations to attribute values to features in the classical RL framework, we therefore reunite concepts of the Pavlovian conditioning and instrumental conditioning literature that are rarely combined together, to model some Pavlovian-instrumental interactions.

One must note that the model of Dayan et al. [4] is also able to replicate the results of the first experiments. It also uses a weighted sum between a classical RL system and some impetus system, and by varying the weight of the two systems, it can also produce behaviours that may be paralleled to sign-tracking and goal-tracking. However, in its current form, their model is unable to reproduce the other experiments of Williams and Williams [8]. Their impetus system is designed to arbitrary bias the model towards an action a priori defined as Pavlovian, in this case *Go* against *NoGo*, by adding the mean reward value of the ongoing experiment. Introducing new alternative *Go* actions raises questions on whether they should be defined as Pavlovian or not, and on the way they should be biased, i.e. using the same mean reward value or a different one. Even so, it seems that this would not explain the preference for intermittent keys versus continuous keys. While there might be ways to make it work, we think that the use of factored representations makes it straightforward and automatic for our model to explain these experimental data and potentially predict how the model would behave in the presence of new stimuli without filling it with a priori informations. The recording of consumption-like engagements towards the magazine during goal-tracking like behaviours would argue in favour of our model, which predicts the acquisition of some motivational value towards the magazine, whereas the model of Dayan et al. [4] does not.

### Resolution of conflicting results

The difference between all pigeons in Williams and Williams [8] but P19 and Sanabria et al. [19] parallels well with the inter-variability observed by Flagel et al. [21] within rats undergoing an autoshaping procedure. In this study, a unique population of rats provided very distinct subgroups. Sign-trackers were prone to engage with the predictive conditioned stimulus (a lever), and goal-trackers were prone to engage with the magazine where food would be delivered as soon as the lever appeared. The computational model reproduces the variability of behaviours in pigeons in these two studies in a similar way, based on the varying influence attributed to each system. The simulated pigeons of Sanabria et al. [19] mainly rely on the MB system, while those of Williams and Williams [8] mainly rely on the FMF system (except for P19). Given the small size of the populations of pigeons involved, one could hope that with a bigger population we could observe within the same study a larger variation of behaviours similar to those of sign-trackers and goal-trackers. Furthermore, it has been shown that populations of rats taken from different vendors (or even different colonies of the same vendor) can show significant differences in their proportion of sign-trackers and goal-trackers [63]. If confirmed in pigeons, such a result could strengthen our hypothesis. This does not discard that part of the difference in the observed behaviours also comes from the difference in protocols between the two studies.

It is interesting to note that in a study about guinea pigs [64], the averaged individual engaged with the conditioned cue under autoshaping phases and switched to engage with the magazine during negative automaintenance phases. Hence, while not engaging with the cue when detrimental, animals could redirect their engagement impulses towards the magazine, in a manner similar to goal-trackers [21]. Such a behaviour could easily be explained by the model with the appropriate parameters, i.e. a reasonably high 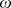 with a low 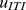. It would be interesting to know if pigeons in which negative automaintenance is effective would do the same, i.e. whether they would redirect their pecks towards the magazine, if made possible (e.g. no blocking door).

Gamzu and Schwam [65] studied negative automaintenance in 4 squirrel monkeys and showed that only one did express a persistent detrimental engagement, and only during early negative automaintenance sessions. They concluded that the procedure fails to produce maladaptive behaviour in these monkeys. Interestingly, the authors state that while key pressing is virtually eliminated, monkeys orient towards the key and occasionally approach it without contact. The model would be able to account for such behaviour with the motivational value of the key sufficiently high to favour approaches towards it rather than the magazine but not high enough so that it would be impossible to refrain from engaging with it. Gamzu and Schwam [65] discuss the fact that, contrary to pigeons, the action of key pressing in monkeys is very different from their consumption behaviour, which could be one of the reason of the failure of the negative automaintenance procedure [66]. Another interpretation, based on the present model, would be that the 4 monkeys are mainly goal-trackers. It might be also the case that monkeys and human brains offer a higher level of control in the integration of the two systems.

### Predictions

**Figure 8:**
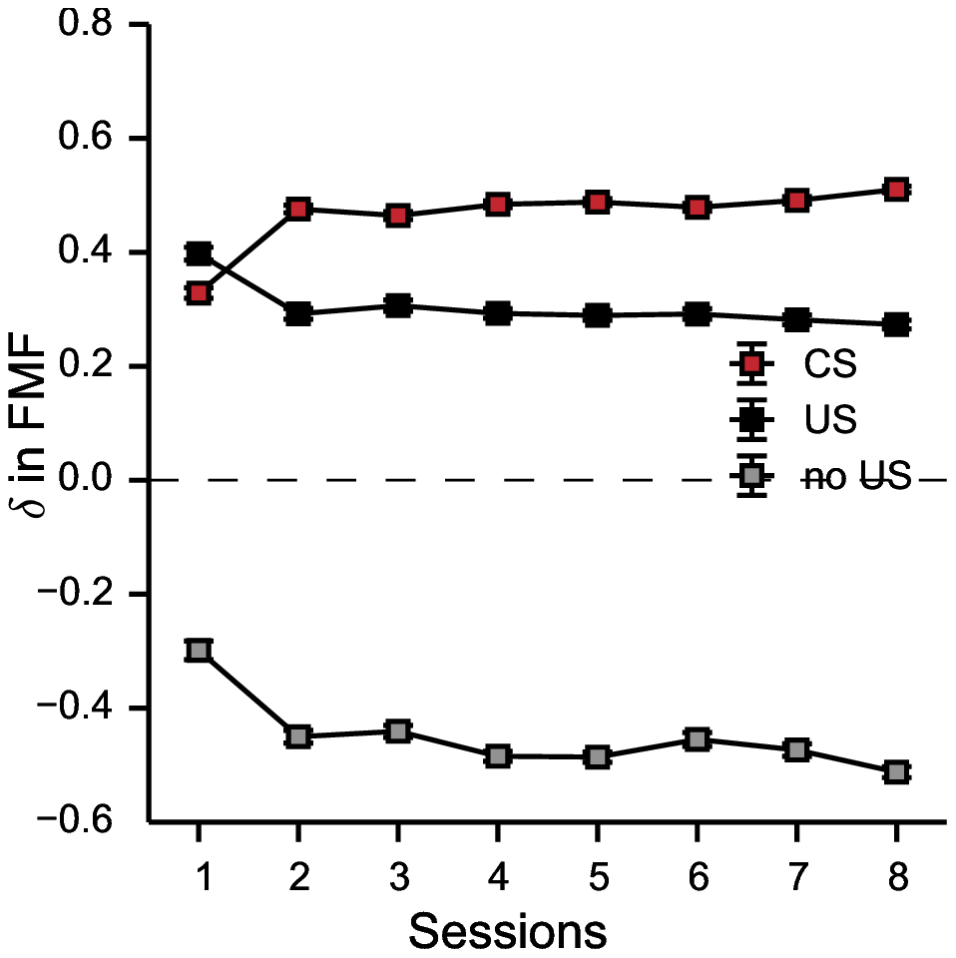
Prediction of the model about expected patterns of dopaminergic activity in negative automaintenance. Data are expressed as mean ± SEM. Average RPE computed by the FMF system at CS appearance (red) and removal of the CS after engagement with the negative key light (no US; gray) and withholding (US; black) for each session of conditioning in the whole population of pigeons (STs and GTs).

One of the motivations behind the development of the computational model of Lesaint et al. [20] was to provide an explanation for the particular patterns of DA recordings observed in rats undergoing an autoshaping procedure [21], which challenged the classical reward prediction error hypothesis [27], [67]. Assuming that some of the dopaminergic pathways in pigeons share a similar role to those of rats [68], the computational model gives predictions about what could be expected from physiological recordings in a negative automaintenance procedure (Figure 8).

The model predicts that in trials where pigeons orient towards the negative key light (STs or GTs confounded) one should observe DA peaks at CS presentation (as classically expected in such experiments [27]). If pigeons refrain from pecking, one should also observe DA peaks at reward delivery, but with a smaller amplitude (i.e. not a full propagation of DA peaks from the US to the CS as would be expected in an autoshaping experiment). Finally, if pigeons peck the negative key light, one should observe a deep in DA activity when the key light is turned-off and no reward is delivered as expected by the classical omission of an anticipated reward. Note that the model does not use an asymmetrical representation of RPEs, hence it might be possible that DA recordings at pecks might not exactly fit the current prediction [69].

Furthermore, the model heavily relies on the hypothesis that the presence of a stimulus, e.g. continuous key light or magazine, during ITI necessarily reduces its value in the FMF system [20], [33]. Hence, the model predicts that changing the experimental protocol for the ITI part could have some impact on the observed pecks. Indeed, we expect that removing the magazine during ITI, e.g. by blocking it by a door, might make it more attractive to pigeons during key light presentation and hence reduce their detrimental pecks towards any negative key light.

In addition, given that RPEs of the FMF system parallel DA recordings within the core of the nucleus accumbens in rats, we can hypothesize the results of possible lesions or inactivation of the homologue of the dopaminergic system in pigeons. We expect that disabling the FMF system would block any consumption-like behaviour, i.e. pecks towards key lights or magazine. We also expect that pigeons that usually favour approach and engagement towards the key lights will shift their behaviour towards a somewhat more erratic one, i.e. engaging the magazine more often than key lights. Finally, the difference of approach and engagement towards negative, irrelevant and continuous key lights should vanish.

### Limitations

As evoked in Lesaint et al. [20], while using factored representations, and making use of the features within particular states, our approach still relies on the discrete time state paradigm of classical RL, where updates are made at regular intervals and assuming no time required for decisions to be taken. This simplification is sufficient to explain the set of data considered here, however it cannot explain the latencies of responses recorded by Williams and Williams [8]. It also prevents us from attempting to qualitatively account for other results of Sanabria et al. [19], given that time is an important factor of their protocols.

Model-Based capacities of rats have been assessed in multiple studies, however such capacities in pigeons remain to be confirmed. Miyata and Fujita [70] showed that pigeons are able to plan one to two steps ahead in mazes, which would confirm their ability to store models of tasks, if simple enough. Further experiments should be conducted to confirm the presence of an MB system in pigeons. Note however that, while the presence of an MB system is necessary to account for the pharmacological data of Flagel et al. [21], there is no experimental data on negative automaintenance that requires its presence. A classical MF system would have provided similar results, as both algorithms eventually converge to the same values.

The current results rely on parameters that are hand tuned and could benefit from exhaustive raw data. While we are able to reproduce tendencies and to explain which mechanisms of the model are responsible for them, we could benefit from data on which to actually fit the model more closely, for example by individual trial-by-trial analyses [71]. Additionally, as done by Flagel et al. [21], a study that combines not only behavioural data but also physiological and pharmacological data could be of great interest in confirming the model, as previously done by Lesaint et al. [20].

We did not focus on pretraining conditions and the impact they have on the resulting behaviours. The only possibility offered by the model resides in its initialisation. As in most reinforcement learning studies, with sufficient time, the current model should eventually converge towards a solution that is independent of initial conditions, which is definitely in discrepancy with what was observed. Especially, data tend to show that pigeons need some time to consider pecking, as if some kind of threshold needed to be reached beforehand. The model does not model such aspects of the tasks.

Finally, we did not discuss possible anatomical counterparts of the systems in our computational model, as the involved experiments did not imply any lesions or pharmacological manipulations, e.g. injections of antagonists of the dopamine. Therefore, at the current stage, it would be highly speculative to define which regions of the pigeon brain can be paralleled to each system.

### Concluding remarks

Here we used an existing computational model to account for different properties of negative automaintenance, a suggested Pavlovian and instrumental interaction phenomenon. This model was initially developed to account for the variability of behaviours observed in autoshaping experiments [20]. Interestingly, the account of both autoshaping and negative automaintenance phenomena relies on two major concepts of the model: Dual learning systems and the use of factored representations to use values over features. This works adds to an emerging set of studies suggesting the presence and collaboration of multiple RL systems in the brain. It questions the classical paradigm of state representations and suggests that further investigation of factored representations in RL models of Pavlovian and instrumental processes experiments may be useful to explain their interactions.

## References

[1] Breland K, Breland M (1961) The misbehavior of organisms. Am Psychol 16: 681.

[2] Hershberger WA (1986) An approach through the looking-glass. Anim Learn Behav 14: 443–451.

[3] Guitart-Masip M, Huys QJM, Fuentemilla L, Dayan P, Duzel E, et al (2012) Go and no-go learning in reward and punishment: interactions between affect and effect. Neuroimage 62: 154–166. 22548809 10.1016/j.neuroimage.2012.04.024 PMC3387384

[4] Dayan P, Niv Y, Seymour B, Daw ND (2006) The misbehavior of value and the discipline of the will. Neural Netw 19: 1153–1160. 16938432 10.1016/j.neunet.2006.03.002

[5] Redish AD, Jensen S, Johnson A (2008) A unified framework for addiction: vulnerabilities in the decision process. Behav Brain Sci 31: 415–437. 18662461 10.1017/S0140525X0800472X PMC3774323

[6] Beierholm UR, Dayan P (2010) Pavlovian-instrumental interaction in observing behavior. PLoS Comput Biol 6: e1000903. 20838580 10.1371/journal.pcbi.1000903 PMC2936515

[7] Clark JJ, Hollon NG, Phillips PEM (2012) Pavlovian valuation systems in learning and decision making. Curr Opin Neurobiol 22: 1054–1061. 22749132 10.1016/j.conb.2012.06.004 PMC3465491

[8] Williams DR, Williams H (1969) Auto-maintenance in the pigeon: Sustained pecking despite contingent non-reinforcement. J Exp Anal Behav 10.1901/jeab.1969.12-511 PMC1338642 16811370

[9] Skinner BF (1938) The behavior of organisms: An experimental analysis. Appleton-Century-Crofts New York, 82–82 pp.

[10] Brown PL, Jenkins HM (1968) Auto-shaping of the pigeon's key peck. J Exp Anal Behav 11: 1–8. 5636851 10.1901/jeab.1968.11-1 PMC1338436

[11] Deich JD, Wasserman EA (1977) Rate and temporal pattern of key pecking under autoshaping and omission schedules of reinforcement. J Exp Anal Behav 27: 399–405. 16812002 10.1901/jeab.1977.27-399 PMC1333604

[12] Griffin RW, Rashotte ME (1973) A note on the negative automaintenance procedure. Bull Psychon Soc 2: 402–404.

[13] Killeen PR (2003) Complex dynamic processes in sign tracking with an omission contingency (negative automaintenance). J Exp Psychol Anim Behav Process 29: 49. 12561133 10.1037/0097-7403.29.1.49 PMC2643130

[14] Woodard WT, Ballinger JC, Bitterman M (1974) Autoshaping: further study of “negative automaintenance”. J Exp Anal Behav 22: 47–51. 16811786 10.1901/jeab.1974.22-47 PMC1333240

[15] Locurto C, Terrace H, Gibbon J (1976) Autoshaping, random control, and omission training in the rat. J Exp Anal Behav 26: 451–462. 16811960 10.1901/jeab.1976.26-451 PMC1333535

[16] Locurto C, Terrace H, Gibbon J (1978) Omission training (negative automaintenance) in the rat: Effects of trial offset. Bull Psychon Soc 12: 11–14.

[17] O'Connell MF (1979) Temporal distributions of responding during discrete-trial omission training in rats. J Exp Anal Behav 31: 31. 16812121 10.1901/jeab.1979.31-31 PMC1332787

[18] Gormezano I, Hiller GW (1972) Omission training of the jaw-movement response of the rabbit to a water us. Psychon Sci 29: 276–278.

[19] Sanabria F, Sitomer MT, Killeen PR (2006) Negative automaintenance omission training is effective. J Exp Anal Behav 86: 1–10. 16903489 10.1901/jeab.2006.36-05 PMC1601948

[20] Lesaint F, Sigaud O, Flagel SB, Robinson TE, Khamassi M (2014) Modelling individual differences in the form of pavlovian conditioned approach responses: A dual learning systems approach with factored representations. PLoS Comput Biol 10: e1003466. 24550719 10.1371/journal.pcbi.1003466 PMC3923662

[21] Flagel SB, Clark JJ, Robinson TE, Mayo L, Czuj A, et al (2011) A selective role for dopamine in stimulus-reward learning. Nature 469: 53–57. 21150898 10.1038/nature09588 PMC3058375

[22] Boakes R (1977) Performance on learning to associate a stimulus with positive reinforcement. Operant-Pavlovian interactions: 67–97.

[23] Dickinson A, Balleine B (1994) Motivational control of goal-directed action. Anim Learn Behav 22: 1–18.

[24] Graybiel AM (2008) Habits, rituals, and the evaluative brain. Annu Rev Neurosci 31: 359–387. 18558860 10.1146/annurev.neuro.29.051605.112851

[25] Dolan RJ, Dayan P (2013) Goals and habits in the brain. Neuron 80: 312–325. 24139036 10.1016/j.neuron.2013.09.007 PMC3807793

[26] Sutton RS, Barto AG (1998) Reinforcement learning: An introduction. The MIT Press.

[27] Schultz W (1998) Predictive reward signal of dopamine neurons. J Neurophysiol 80: 1–27. 9658025 10.1152/jn.1998.80.1.1

[28] Niv Y (2009) Reinforcement learning in the brain. J Math Psychol 53: 139–154.

[29] Mahler SV, Berridge KC (2009) Which cue to “want?” Central amygdala opioid activation enhances and focuses incentive salience on a prepotent reward cue. J Neurosci 29: 6500–13. 19458221 10.1523/JNEUROSCI.3875-08.2009 PMC2802210

[30] DiFeliceantonio AG, Berridge KC (2012) Which cue to ‘want’? Opioid stimulation of central amygdala makes goal-trackers show stronger goal-tracking, just as sign-trackers show stronger sign-tracking. Behav Brain Res 230: 399–408. 22391118 10.1016/j.bbr.2012.02.032 PMC3322261

[31] Berridge KC (2007) The debate over dopamines role in reward: the case for incentive salience. Psychopharmacology 191: 391–431. 17072591 10.1007/s00213-006-0578-x

[32] Guitart-Masip M, Duzel E, Dolan R, Dayan P (2014) Action versus valence in decision making. Trends Cogin Sci 18: 194–202. 10.1016/j.tics.2014.01.003 PMC3989998 24581556

[33] Lesaint F, Sigaud O, Clark JJ, Flagel SB, Khamassi M (2014) Experimental predictions drawn from a computational model of sign-trackers and goal-trackers. J Physiol Paris: in press. 10.1016/j.jphysparis.2014.06.001 PMC4272685 24954026

[34] Dayan P, Berridge KC (2014) Model-based and model-free pavlovian reward learning: Revaluation, revision, and revelation. Cogn Affect Behav Neurosci: 1–20. 10.3758/s13415-014-0277-8 PMC4074442 24647659

[35] Dayan P, Balleine BW (2002) Reward, motivation, and reinforcement learning. Neuron 36: 285–298. 12383782 10.1016/s0896-6273(02)00963-7

[36] Nicola SM (2010) The flexible approach hypothesis: unification of effort and cue-responding hypotheses for the role of nucleus accumbens dopamine in the activation of reward-seeking behavior. J Neurosci 30: 16585–16600. 21147998 10.1523/JNEUROSCI.3958-10.2010 PMC3030450

[37] Huys QJM, Cools R, Gölzer M, Friedel E, Heinz A, et al (2011) Disentangling the roles of approach, activation and valence in instrumental and pavlovian responding. PLoS Comput Biol 7: e1002028. 21556131 10.1371/journal.pcbi.1002028 PMC3080848

[38] Geurts DE, Huys QJ, den Ouden HE, Cools R (2013) Aversive pavlovian control of instrumental behavior in humans. J Cogn Neurosci 25: 1428–1441. 23691985 10.1162/jocn_a_00425

[39] Yin HH, Ostlund SB, Balleine BW (2008) Reward-guided learning beyond dopamine in the nucleus accumbens: the integrative functions of cortico-basal ganglia networks. Eur J neurosci 28: 1437–1448. 18793321 10.1111/j.1460-9568.2008.06422.x PMC2756656

[40] Alloy LB, Ehrman RN (1981) Instrumental to pavlovian transfer: Learning about response-reinforcer contingencies affects subsequent learning about stimulus-reinforcer contingencies. Learn Motiv 12: 109–132.

[41] Prévost C, McNamee D, Jessup RK, Bossaerts P, O'Doherty JP (2013) Evidence for model-based computations in the human amygdala during pavlovian conditioning. PLoS Comput Biol 9: e1002918. 23436990 10.1371/journal.pcbi.1002918 PMC3578744

[42] Balleine BW, O'Doherty JP (2009) Human and rodent homologies in action control: corticostriatal determinants of goal-directed and habitual action. Neuropsychopharmacology 35: 48–69. 10.1038/npp.2009.131 PMC3055420 19776734

[43] Daw ND, Niv Y, Dayan P (2005) Uncertainty-based competition between prefrontal and dorsolateral striatal systems for behavioral control. Nat Neurosci 8: 1704–1711. 16286932 10.1038/nn1560

[44] Keramati M, Dezfouli A, Piray P (2011) Speed/Accuracy trade-off between the habitual and the goal-directed processes. PLoS Comput Biol 7: e1002055. 21637741 10.1371/journal.pcbi.1002055 PMC3102758

[45] Pezzulo G, Rigoli F, Chersi F (2013) The mixed instrumental controller: using value of information to combine habitual choice and mental simulation. Front Psychol 4 10.3389/fpsyg.2013.00092 PMC3586710 23459512

[46] Meyer PJ, Lovic V, Saunders BT, Yager LM, Flagel SB, et al (2012) Quantifying individual variation in the propensity to attribute incentive salience to reward cues. PLoS ONE 7: e38987. 22761718 10.1371/journal.pone.0038987 PMC3382216

[47] Corbit LH, Balleine BW (2005) Double dissociation of basolateral and central amygdala lesions on the general and outcome-specific forms of pavlovian-instrumental transfer. J Neurosci 25: 962–970. 15673677 10.1523/JNEUROSCI.4507-04.2005 PMC6725628

[48] Schmajuk NA, Lam YW, Gray JA (1996) Latent inhibition: A neural network approach. J Exp Psychol Anim Behav Process 22: 321–349. 8691162 10.1037//0097-7403.22.3.321

[49] Balkenius C (1999) Dynamics of a classical conditioning model. Auton Robots 7: 41–56.

[50] Redish AD, Jensen S, Johnson A, Kurth-Nelson Z (2007) Reconciling reinforcement learning models with behavioral extinction and renewal: Implications for addiction, relapse, and problem gambling. Psychol Rev 114: 784–805. 17638506 10.1037/0033-295X.114.3.784

[51] Stout SC, Miller RR (2007) Sometimes-competing retrieval (SOCR): A formalization of the comparator hypothesis. Psychol Rev 114: 759–783. 17638505 10.1037/0033-295X.114.3.759

[52] Courville AC, Daw ND, Touretzky DS (2006) Bayesian theories of conditioning in a changing world. Trends Cogn Sci 10: 294–300. 16793323 10.1016/j.tics.2006.05.004

[53] Gershman SJ, Niv Y (2012) Exploring a latent cause theory of classical conditioning. Anim Learn Behav 40: 255–268. 10.3758/s13420-012-0080-8 22927000

[54] Kamin LJ (1967) Predictability, surprise, attention, and conditioning. In: Campbell BA, Church RMa, editors, Punishment and aversive behavior, New York: Appleton-Century-Crofts. pp.279–296.

[55] Lattal KM, Nakajima S (1998) Overexpectation in appetitive pavlovian and instrumental conditioning. Anim Learn Behav 26: 351–360.

[56] Doya K, Samejima K, Katagiri Ki, Kawato M (2002) Multiple model-based reinforcement learning. Neural Comput 14: 1347–1369. 12020450 10.1162/089976602753712972

[57] Bellman R (1957) Dynamic programming. Princeton University Press.

[58] Khamassi M, Martinet LE, Guillot A (2006) Combining self-organizing maps with mixtures of experts: application to an actor-critic model of reinforcement learning in the basal ganglia. In: From Animals to Animats 9, Springer. pp.394–405.

[59] Elfwing S, Uchibe E, Doya K (2013) Scaled free-energy based reinforcement learning for robust and efficient learning in high-dimensional state spaces. Front Neurorobot 7 10.3389/fnbot.2013.00003 PMC3584292 23450126

[60] Boutilier C, Dearden R, Goldszmidt M (2000) Stochastic dynamic programming with factored representations. Artif Intell 121: 49–107.

[61] Degris T, Sigaud O, Wuillemin PH (2006) Learning the structure of factored markov decision processes in reinforcement learning problems. In: Proceedings of the 23rd international conference on Machine learning. ACM, pp.257–264.

[62] Vigorito CM, Barto AG (2008) Autonomous hierarchical skill acquisition in factored mdps. In: Yale Workshop on Adaptive and Learning Systems, New Haven, Connecticut.

[63] Fitzpatrick CJ, Gopalakrishnan S, Cogan ES, Yager LM, Meyer PJ, et al (2013) Variation in the form of pavlovian conditioned approach behavior among outbred male sprague-dawley rats from different vendors and colonies: Sign-tracking vs. goal-tracking. PloS ONE 8: e75042. 24098363 10.1371/journal.pone.0075042 PMC3787975

[64] Poling A, Poling T (1978) Automaintenance in guinea pigs: Effects of feeding regimen and omission training. J Exp Anal Behav 30: 37–46. 16812086 10.1901/jeab.1978.30-37 PMC1332730

[65] Gamzu E, Schwam E (1974) Autoshaping and automaintenance of a key-press response in squirrel monkeys. J Exp Anal Behav 21: 361–371. 16811749 10.1901/jeab.1974.21-361 PMC1333204

[66] Meyer PJ, Cogan ES, Robinson TE (2014) The form of a conditioned stimulus can influence the degree to which it acquires incentive motivational properties. PloS ONE 9: e98163. 24905195 10.1371/journal.pone.0098163 PMC4048203

[67] Fiorillo CD, Tobler PN, Schultz W (2003) Discrete coding of reward probability and uncertainty by dopamine neurons. Science 299: 1898–1902. 12649484 10.1126/science.1077349

[68] Gargiulo PA, Acerbo MJ, Krug I, Delius J (2005) Cognitive effects of dopaminergic and glutamatergic blockade in nucleus accumbens in pigeons. Pharmacology Biochemistry and Behavior 81: 732–739. 10.1016/j.pbb.2005.05.009 15979133

[69] Niv Y, Duff MO, Dayan P (2005) Dopamine, uncertainty and td learning. Behavioral and Brain Functions 1: 1–9. 15953384 10.1186/1744-9081-1-6 PMC1171969

[70] Miyata H, Fujita K (2008) Pigeons (columba livia) plan future moves on computerized maze tasks. Anim Cogn 11: 505–516. 18256862 10.1007/s10071-008-0141-8

[71] Daw ND (2011) Trial-by-trial data analysis using computational models. In: Delgado MR, Phelps EA, Robbins TW, editors, Decision Making, Affect, and Learning: Attention and Performance XXIII, Oxford University Press, volume 23, chapter 1.

